# Supplementary material for: Revealing roles of S-layer protein (SlpA) in Clostridioides difficile pathogenicity by generating the first slpA gene deletion mutant
Source: Microbiol Spectr. 2024 May 6;12(6):e04005-23. doi: 10.1128/spectrum.04005-23 (PMC11237437; doi:10.1128/spectrum.04005-23)
Supplement: Supplemental figures — Fig. S1 and S2. [file spectrum.04005-23-s0001.docx]

**Supplemental materials**

**Revealing roles of S-layer protein (SlpA) in *Clostridioides difficile* pathogenicity by generating the first *SlpA* gene knockout mutant**

Shaohua Wang^1,2^*, Maria C Courreges^1^, Lingjun Xu^3^, Bijay Gurung^1^, Mark Berryman^1^, Tingyue Gu^3^

^1^Department of Biomedical Sciences, Ohio University Heritage College of Osteopathic Medicine, Ohio University, Athens, OH, 45701 USA

^2^Infectious and Tropical Disease Institute, Ohio University, Athens, OH, 45701 USA

^3^Department of Chemical and Biomolecular Engineering, Institute for Corrosion and Multiphase Technology, Ohio University, Athens, OH, 45701 USA

***Corresponding author:**

Shaohua Wang, Ph.D.

Department of Biomedical Sciences, Ohio University Heritage College of Osteopathic Medicine, Ohio University

Address: 7 Depot St., Ohio University, Athens, OH, USA 45701

Email: wangs4@ohio.edu

Phone: 1-740-593-2355

Fax: 1-740-597-2778

**Fig. S1**
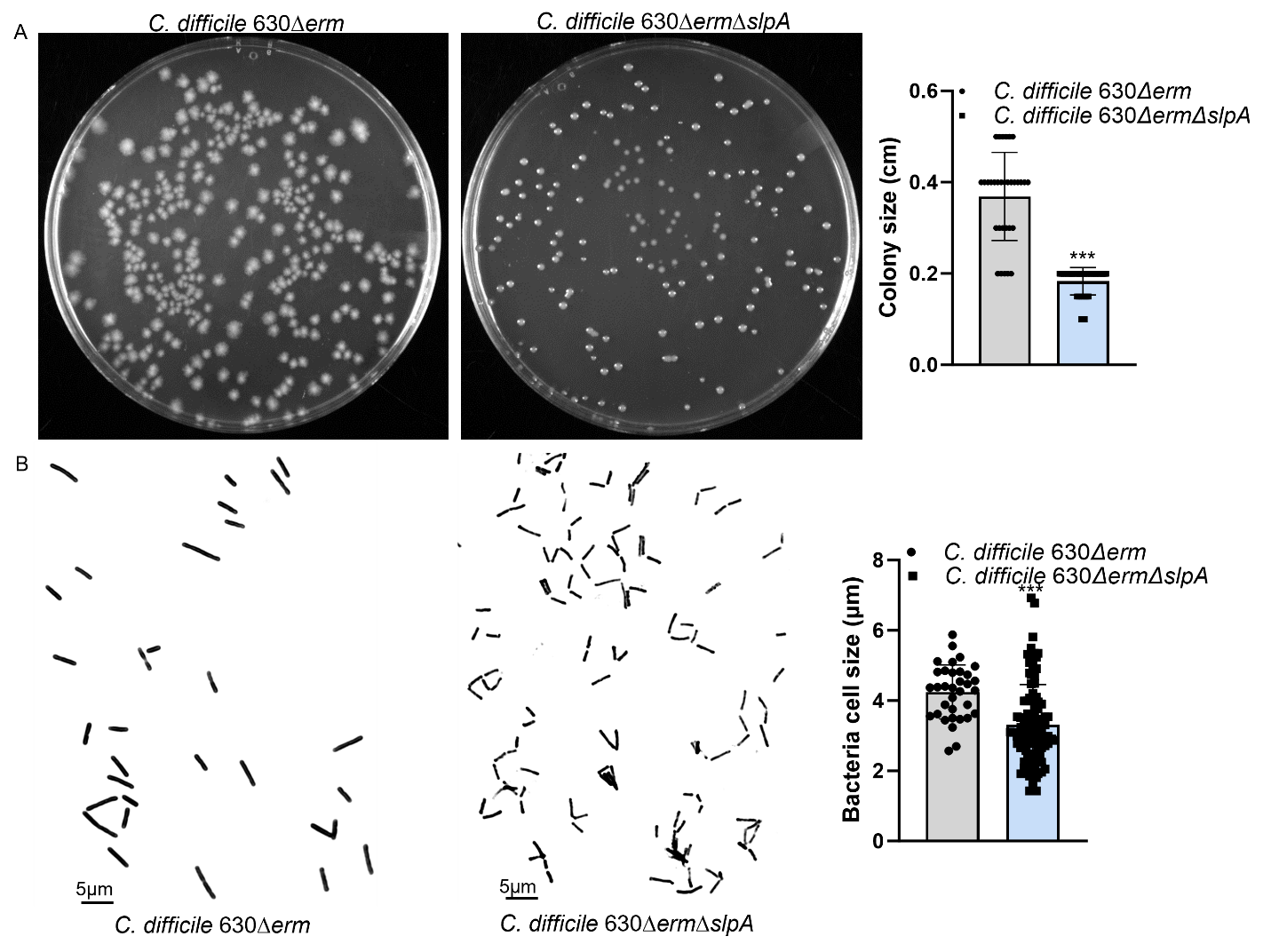


Figure S1. Effects of SlpA on morphology of *C. difficile*. (A) *C. difficile* 630*∆erm∆slpA* showed smaller colony sizes than the wild type *C. difficile* 630*∆erm* strain. (B) Length of *C. difficile* 630*∆erm∆slpA* was also significantly shorter than the wild type under microscope. P-values for differences between the wild type and mutant strains, *** P<0.001.

**Fig. S2**


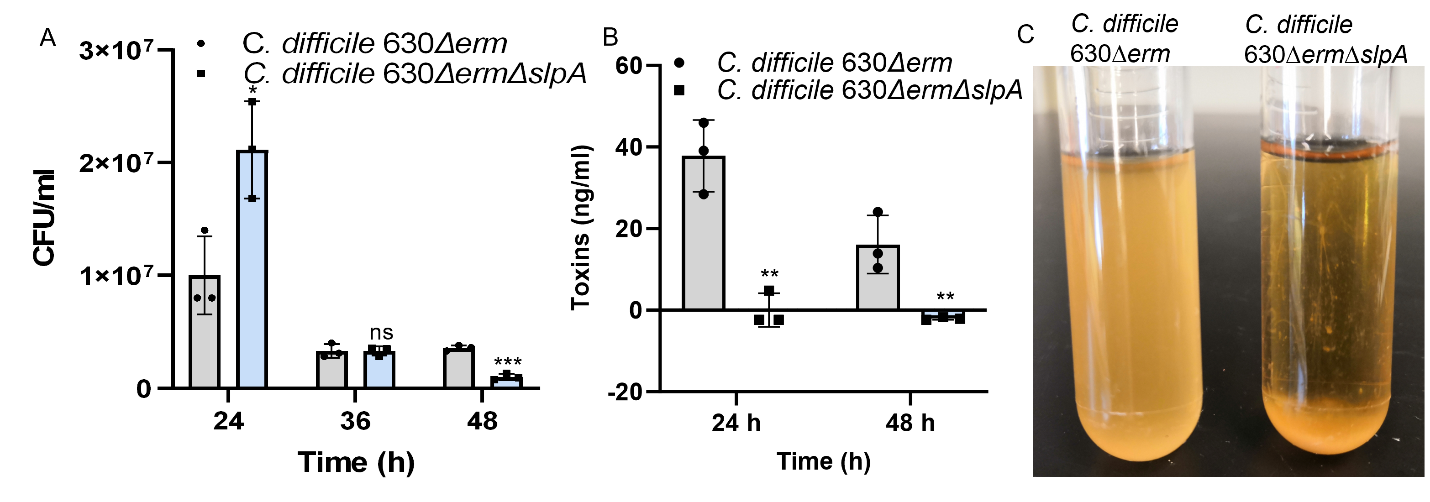


Figure S2. Effects of SlpA on cell concentration (CFU at 24 h, 36 h and 48 h cultivation) (A), toxins production at 24 h and 48 h cultivation (B), and aggregation of *C. difficile* in suspension (C) of *C. difficile*. P-values for differences between the wild type and mutant strains, * P<0.05, ** P<0.01, and *** P<0.001.
